# Non-Cytokine Protein Profile of the Mesenchymal Stem Cell Secretome That Regulates the Androgen Production Pathway

**DOI:** 10.3390/ijms23094633

**Published:** 2022-04-22

**Authors:** Hang-Soo Park, Rishi Man Chugh, Melissa R. Pergande, Esra Cetin, Hiba Siblini, Sahar Esfandyari, Stephanie M. Cologna, Ayman Al-Hendy

**Affiliations:** 1 Department of Obstetrics and Gynecology, University of Chicago, 5841 S. Maryland Ave., Chicago, IL 60637, USA; hspark06@bsd.uchicago.edu (H.-S.P.); ecetin@bsd.uchicago.edu (E.C.); hsiblini@bsd.uchicago.edu (H.S.); 2 Department of Surgery, University of Illinois at Chicago, 820 South Wood Street, Chicago, IL 60612, USA; rchugh@kumc.edu (R.M.C.); sesfan2@uic.edu (S.E.); 3 Department of Radiation Oncology, University of Kansas Medical Center, Kansas City, KS 66160, USA; 4 Department of Chemistry, University of Illinois at Chicago, Chicago, IL 60607, USA; mperga2@uic.edu (M.R.P.); cologna@uic.edu (S.M.C.)

**Keywords:** mesenchymal stem cells, secretome, protein profile, androgen production, polycystic ovary syndrome

## Abstract

Polycystic ovary syndrome (PCOS) is the most common endocrine and metabolic disorder in reproductive-aged women, and it typically involves elevated androgen levels. Recently, it has been reported that human bone marrow mesenchymal stem cells (hBM-MSCs) can regulate androgen synthesis pathways. However, the details of the mechanism are still unclear. hBM-MSC-derived secreted factors (the secretome) are promising sources of cell-based therapy as they consist of various types of proteins. It is thus important to know which proteins interact with disease-implicated biomolecules. This work aimed to investigate which secretome components contain the key factor that inhibits testosterone synthesis. In this study, we fractionated hBM-MSC-conditioned media into three fractions based on their molecular weights and found that, of the three fractions, one had the ability to inhibit the androgen-producing genes efficiently. We also analyzed the components of this fraction and established a protein profile of the hBM-MSC secretome, which was shown to inhibit androgen synthesis. Our study describes a set of protein components present in the hBM-MSC secretome that can be used therapeutically to treat PCOS by regulating androgen production for the first time.

## 1. Introduction

Polycystic ovary syndrome (PCOS) is one of the most common endocrine disorders among reproductive-aged women, leading to chronic low-grade inflammation and excess androgen production by the ovarian theca cells, and even infertility [1,2]. It is reported that around 4 to 18% of reproductive-aged women suffer from PCOS [3]. PCOS is a systemic disease that shows various aberrations. Several studies suggest that PCOS conditions are highly related to ovarian inflammation and stimulated ovarian androgen synthesis, which may even lead to insulin resistance [4,5]. In addition, several published studies have reported that inflammatory cytokines can stimulate androgen-production gene expression, including *CYP11A1*, *CYP17A1* and *3β-hydroxysteroid dehydrogenase* (*HSD3b*) [6,7,8]. Moreover, various studies report a higher risk of type 2 diabetes, cardiovascular diseases and increased endometrial cancer in women with PCOS [9,10,11]. Taken together, treating PCOS is a very important issue to improve women’s health and quality of life. To reverse PCOS, researchers have suggested various therapeutic strategies such as nutritional and anti-inflammatory modulation [12,13,14]. Other recent studies have reported the therapeutic effect of mesenchymal stem cells (MSCs) on PCOS in animal models [15,16]. Further, MSCs’ secretory factors have been reported for their efficacy in treating various disease conditions, such as neurological disorders, cardiac ischemic disorders, diabetes mellitus and liver injury or fibrosis [17,18,19,20].

According to previous studies, the primary mechanism of the therapeutic efficacy of MSCs is mediated by their migration ability toward damaged tissues [21,22,23]. MSCs can also repair damaged tissues in a paracrine manner [24] by secreting various components, such as growth factors, cytokines and extracellular vesicles that exert immunosuppressive, anti-apoptotic, anti-fibrotic, angiogenic and anti-inflammatory effects [24,25,26,27]. Due to these various regulatory potentials, MSCs are a promising candidate for treating PCOS patients. A published study already showed the therapeutic potential of MSCs in a PCOS animal model via their anti-inflammatory properties [15,16]. Interestingly, we also found a similar therapeutic effect of MSCs in our previous unpublished data. To enhance the therapeutic potential of MSCs in PCOS, it is necessary to identify the regulatory mechanism of MSCs and their secretory factors. 

There are several previous studies that have analyzed the protein profile of the MSC secretome [28,29,30]. One study in 2017 reported 961 BM-MSC-derived proteins using mass spectrometry [30]. A more recent study also reported 270 unique proteins found in MSC-derived extracellular vesicles [29]. However, these previous studies demonstrated only the general profile of MSC-derived proteins and provide little insight regarding the mechanism by which MSC secretory factors may modulate PCOS pathology. This study represents the first step towards enhancing the therapeutic efficacy of MSCs for PCOS treatment. Thus far, researchers have hypothesized that the immunosuppressive properties of MSCs could be a key mechanism in reversing PCOS [16], but the detailed pathway is not clear. Given this vast knowledge gap, it is important to identify the key molecules of the MSC secretome that regulate PCOS-related pathology. Additionally, it would be useful to identify those responsible for the suppression of androgen synthesis or anti-inflammatory pathways. With this information, it will be possible to discover a therapeutic modality for PCOS and to understand the therapeutic mechanisms of MSCs-based treatment.

In this study, we analyzed the protein profile of MSC secretomes to identify unique proteins that have a direct effect on reversing PCOS pathology. As the first step, we fractionated MSC-conditioned medium (MSC-CM) based on size using a sephadex column. Next, we analyzed the therapeutic effect of each fraction on PCOS condition by analyzing androgen synthesis genes in in vitro PCOS model H295R cells. This cell line has been used in previous studies as an in vitro model of human PCOS ovarian theca cells [31] and as a model of androgen production [32,33,34]. Another published study also reported that *CYP17A1*, *CYP11A1* and *DENND1A* are key genes for ovarian androgen synthesis and are upregulated in PCOS-theca cells, compared with healthy theca cells [35,36]. In our study, we treated the H295R cells with the MSC-CM fractions individually and measured the above genes to evaluate changes in the androgen production pathway. Based on the results of the in vitro analysis and the mass spectrometry data, we report several unique proteins that may regulate the androgen synthesis pathway in PCOS.

## 2. Results

### 2.1. Effect of Fractionated Sample on Androgen Production

**Figure 1:**
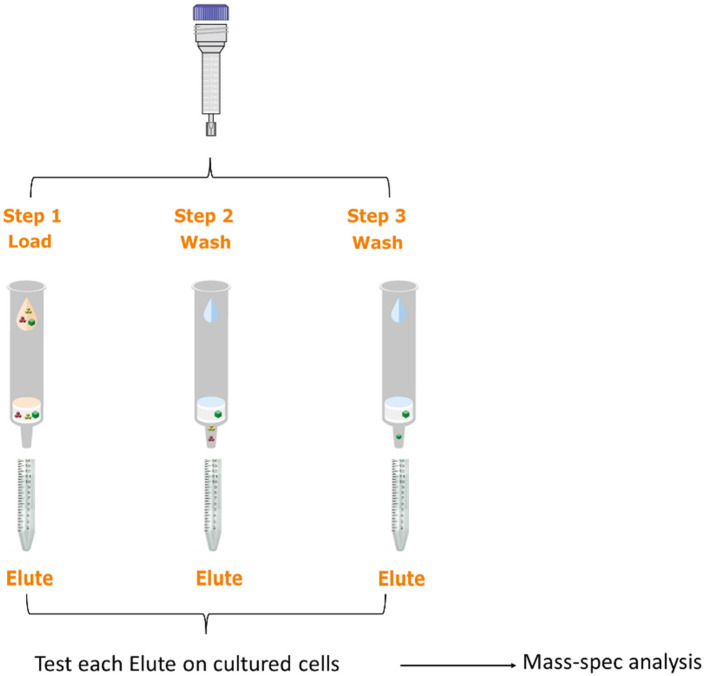
Fractionation of MSC-derived conditioned media. Whole conditioned media were passed through the Sephadex-15 column and we collected the gravity-based elute. Each fraction (Elute) was analyzed by mass spectrometer for further protein profiling.

**Figure 2:**
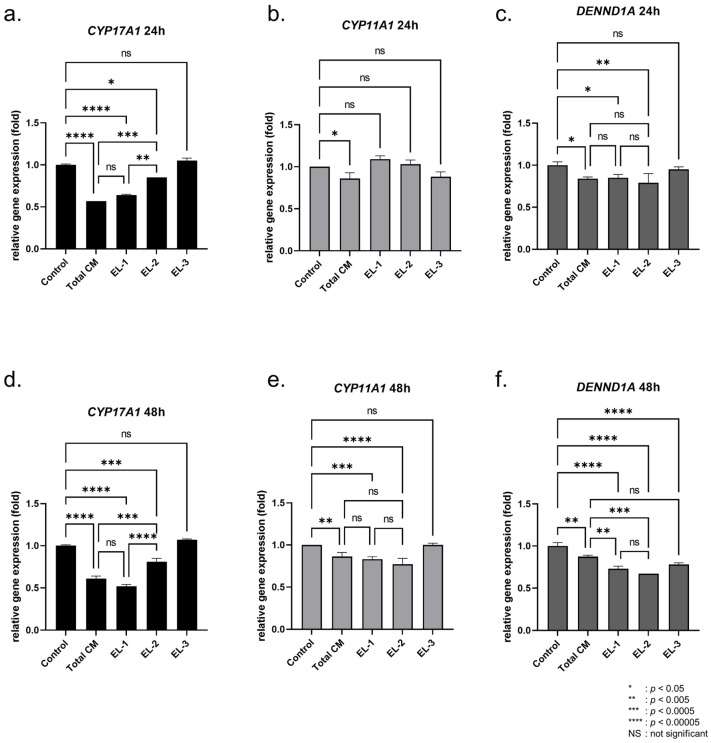
Effect of fractionated sample on androgen production gene expression. RNA expression level of androgen-producing genes in H295R cells after total MSC-CM (Total CM), Elute-1 (EL-1), Elute-2 (EL-2) and Elute-3 (EL-3) treatment. Relative gene expression of *CYP17A1*, *CYP11A1* and *DENND1A* after 24 h of treatment (**a**–**c**). Relative gene expression of *CYP17A1*, *CYP11A1* and *DENND1A* after 48 h of treatment (**d**–**f**). Comparisons between groups were made by two-way ANOVA. Data are presented as the mean ± SD. (*n* = 3, significance level: * *p* < 0.05, ** *p* < 0.005, *** *p* < 0.0005, **** *p* < 0.00005; NS: not significant).

In our previous study, we found that MSC-conditioned medium (MSC-CM) can inhibit androgen production in the androgen producing H295R cell line [37,38]. In this study, we further identified the main components of the androgen regulation pathway in MSC-CM. We collected the MSC-CM 24 and 48 h after being cultured with MSCs. We fractionated the MSC-CM to prepare three different elutes based on protein size using a Sephadex bead-based approach (Figure 1). MSC-CM media were passed through the column and we collected the gravity-based elute (Elute-1, EL-1). In this approach, the first elute contained larger molecules (e.g., large proteins). Next, a second fraction was collected, which presumably contained larger peptides (Elute-2, EL-2). Lastly, a third fraction was collected, which presumably contained smaller peptides and metabolites (Elute-3, EL-3). To analyze the effect of the MSC-CM fractions on the androgen production pathway, we analyzed the RNA expression of the androgen-producing genes *CYP17A1*, *CYP11A1* and *DENND1A* in MSC-CM or elute-treated H295R cells (Figure 2). Here, we diluted the concentrated elute with culture media at a 1:1 ratio prior to H295R cell treatment. After 24 and 48 h of treatment, the cells were collected for further analysis. 

In our RT-qPCR result from the 24 h CM-treated group, we found that the expression of the androgen-producing gene *CYP17A1* was significantly decreased (0.57 ± 0.00-fold) in total MSC-CM (total CM)-treated cells, compared to the untreated control cells. Interestingly, the Elute-1 (EL-1)-treated H295R cells showed significantly decreased *CYP17A1* expression (0.64 ± 0.01-fold), with no significant difference compared to the total CM group (*p* = 0.544). Elute-2 (EL-2) also showed decreased *CYP17A1* expression (0.85 ± 0.00-fold), significantly higher than the total CM group (*p* = 0.0001). Elute-3, however, did not show decreased *CYP17A1* gene expression (1.05 ± 0.03-fold) in the H295R cells (Figure 2a). The expression of *CYP11A1* was decreased in H295R after treatment with total CM (0.86 ± 0.07-fold). However, neither Elute-1, Elute-2 nor Elute-3 alone had significantly decreased *CYP11A1* expression levels (Figure 2b). An evaluation of *DENND1A* gene levels showed decreased expression in the total CM-treated group (0.84 ± 0.02-fold), the EL-1 group (0.85 ± 0.04-fold) and the EL-2 group (0.79 ± 0.11-fold); however, no significant differences between these three groups were observed (Figure 2c).

We also analyzed the expression levels of these three genes in the 48-h CM-treated cells. The total CM group showed significantly decreased expression of *CYP171A* (0.61 ± 0.03-fold) and the EL-1 group also had reduced *CYP17A1* expression (0.52 ± 0.02-fold), similar to that of the total CM group (*p* = 0.079). *CYP17A1* expression was decreased in the EL-2 group (0.81 ± 0.04-fold), but was still higher than that of the total CM group (*p* = 0.0001) (Figure 2d). *CYP11A1* gene expression was decreased in the total CM group (0.86 ± 0.05-fold), EL-1 group (0.83 ± 0.03-fold) and EL-2 group (0.77 ± 0.07-fold), but no significant difference was observed between the three groups (Figure 2e). *DENND1A* expression was significantly decreased in all groups. Interestingly, *DENND1A* expression in the EL-1 group (0.73 ± 0.03-fold) and EL-2 group (0.67 ± 0.00-fold) showed even greater decreases than in the total CM-treated group (0.87 ± 0.02-fold) (Figure 2f). There was no significant difference in DENND1A expression between the EL-1 group and EL-2 group. Taken together, EL-1 regulates all three genes’ expression (*CYP17A1*, *CYP11A1* and *DENND1A*), whereas EL-2 regulates only two genes (*CYP11A1* and DENND1A) and EL-3 regulates only DENND1A.

Our data demonstrate that total CM inhibits androgen production pathway gene expression, and EL-1 shows the most similar regulatory effect on androgen-producing genes among the three fraction samples. In the subsequent assay, we analyzed the protein components of EL-1 to identify proteins that may be responsible for the inhibition of the androgen production pathway. 

### 2.2. Identification of MSC Proteins That Regulate Androgen Synthesis

**Figure 3:**
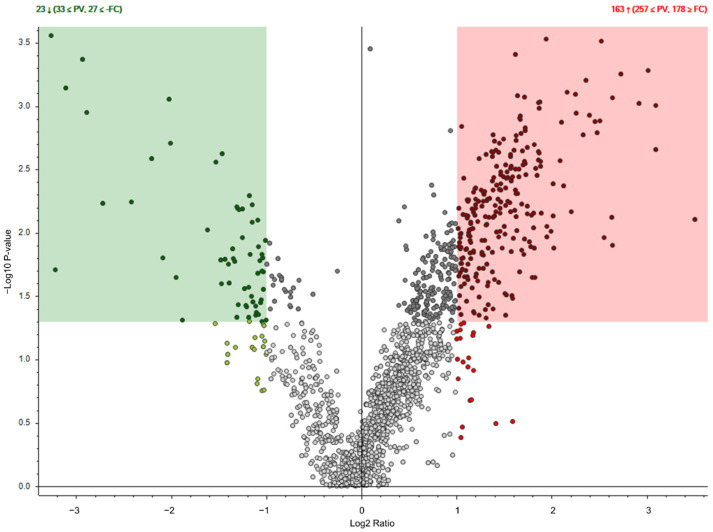
Identification of specific components secreted by MSC. Volcano plot comparison analysis of MSC-CM and fibroblast-conditioned media. Among 893 identified proteins in MSC-CM, 178 proteins show more than two-fold higher abundance in MSC-CM compared to control fibroblast CM.

**Figure 4:**
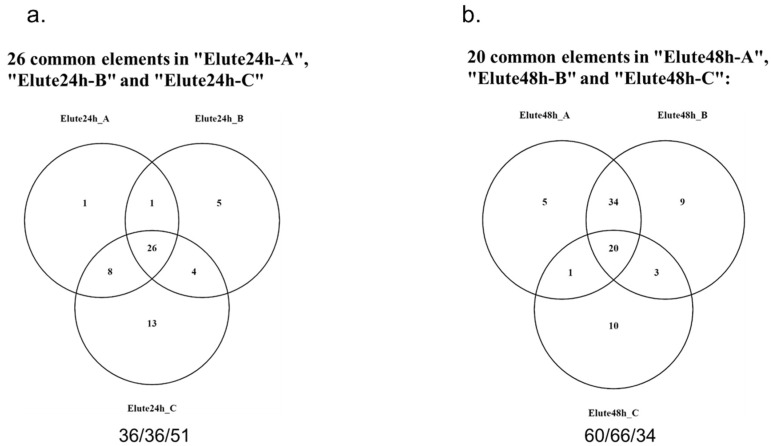
Overlap of proteins identified in MSC-CM EL-1. The EL-1 fractions for three independent MSC-CM samples were analyzed. (**a**) Venn diagram of three independent EL-1 fractions secreted by MSC for 24 h. Here, 26 proteins were detected in all three independent samples (Table 1). (**b**) Venn diagram of three independent EL-1 fractions secreted by MSC for 48 h. Here, 20 proteins were detected in all three independent samples (Table 2).

To identify specific components of MSC-CM, relative protein levels in the total MSC-CM were subject to analysis via liquid chromatography–mass spectrometry (LC–MS), following which the relative protein levels were compared with those of CM from fibroblasts. In our data, we found 893 identified proteins in the MSC-CM. Among these candidate proteins, 178 showed more than two-fold higher abundance in the MSC-CM compared to the control fibroblast CM. (Figure 3, Supplementary Table S1). Based on our analysis of *CYP17A1*, *CYP11A1* and *DENND1A*, we confirmed that EL-1 contains the most factors that inhibit the above androgen-producing genes. To minimize candidate pathways, we further analyzed the MSC-CM fraction EL1 to identify proteins that might influence androgen production gene expression. To minimize batch-to-batch variation, we prepared three independent samples of EL-1. In the 24-h samples (Elute24h), we identified 36, 36 and 51 proteins in Elute24h_A, Elute24h_B and Elute24h_C, respectively. Among these proteins, we found 26 common proteins in all the Elute24h samples (Figure 4a and Table 1). In the 48-h samples (Elute48h), we identified 60, 66 and 34 proteins in Elute48h_A, Elute48h_B and Elute48h_C, respectively. Among these proteins, we found 20 common proteins in all the Elute48h samples (Figure 4b and Table 2). In addition, we found that 13 proteins (ACTB, A1BG, AHSG, APOA1, COL1A1, COL1A2, FN1, HP, HPX, ITIH4, PGLYRP2, TF and ALB) were common in both Elute24h and Elute48h, as well as 13 unique proteins in Elute24 (APOH, FBN2, FBLN1, FBLN5, HP, HBA1, IGHG3, IGFBP4, IGFBP6, KNG1, LTBP2, TIMP1 and THBS2) and 7 unique proteins in Elute48h (AFM, CLU, COL3A1, COL6A1, COL5A2, DCN and GAS6). 

### 2.3. Regulation of Androgen Synthesis Genes by MSC-Derived Proteins

**Figure 5:**
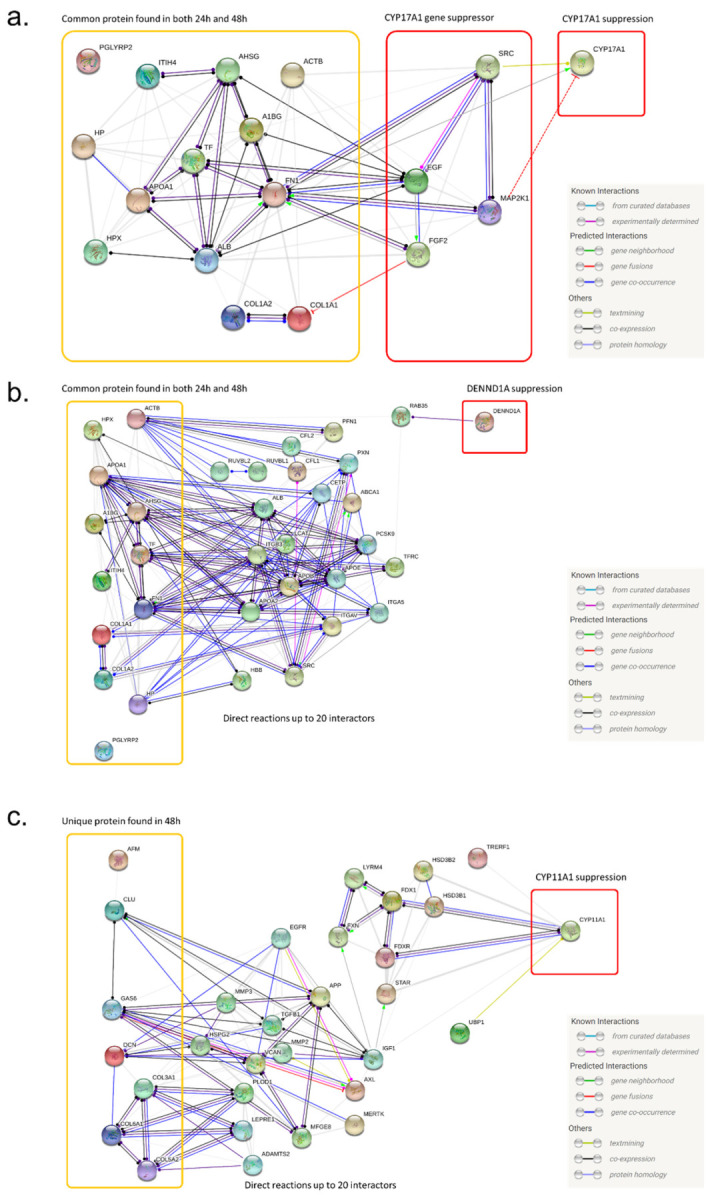
Expected regulatory pathway of androgen synthesis genes by MSC-derived proteins. The protein interaction network for *CYP17A1*, *DENND1A* and *CYP11A1*, according to the database test mining algorithm (STRING). (**a**) Predicted regulation of *CYP17A1* through the MAPK (MEK) pathway by MSC secreted proteins. (**b**) Predicted regulation of *DENND1A* expression in the 20 most promising direct protein interactions with MSC-secreted proteins. (**c**) Predicted regulation of *CYP11A1* expression through the 20 most promising direct protein interactions with MSC-secreted proteins.

Based on our RT-qPCR and proteomics data, we predicted a regulation pathway for androgen-producing genes, specifically *CYP17A1*, *CYP11A1* and *DENND1A*, by MSC-CM-derived proteins. Based on the database test mining algorithm STRING, we created a protein interaction network for each gene. To predict a pathway that could explain our RT-qPCR data, we searched the direct interactions between proteins from the MSC-CM (EL-1 identified proteins) and target genes. For *CYP17A1*, we found that all 13 common proteins found in both Elute24h and Elute 48h (ACTB, A1BG, AHSG, APOA1, COL1A1, COL1A2, FN1, HP, HPX, ITIH4, PGLYRP2, TF and ALB) had evidence of direct interaction with the SRC (proto-oncogene tyrosine-protein kinase) pathway and MAPK (MEK) pathway (Figure 5a). In a published study, it was reported that the SRC and MEK pathways suppress *CYP17A1* gene expression in H295R cells. Taken together, we suggest a predicted regulation pathway that suppresses the *CYP17A1* gene via 13 common proteins derived from MSC-CM.

Using a similar method, we also built a protein interaction network for *DENND1A* and *CYP11A1*. Unfortunately, we could not find a direct interaction between the 13 common proteins and *DENND1A* or a suppressor of *DENND1A*. However, we found that there were various potential candidate regulators of *DENND1A*, which may be a link between the regulation pathway of 13 common proteins and *DENND1A* (Figure 5b). For *CYP11A1*, we used another protein profile based on the PCR result using Elute48h. These seven unique proteins (AFM, CLU, COL3A1, COL6A1, COL5A2, DCN and GAS6) were found only in Elute48 and were absent in Elute24h. Like the *DENND1A* suppression pathway, there was no direct interaction between the seven unique proteins and the *CYP11A1* protein. Instead, we reported various potential candidate regulators that may be a link between the regulation pathway, the seven unique proteins and *CYP11A1* (Figure 5c). Taken together, we suggest an expected suppression pathway of androgen-producing genes such as *CYP17A1*, *CYP11A1* and *DENND1A* via MSC-CM-derived proteins.

## 3. Discussion

In this study, we analyzed the protein profile of the MSC secretome using mass spectrometry. Using the fractionation method, a size-based fractionation of components in the secretome was analyzed and an activity assay was performed on a cellular model of PCOS H295R cells. We listed the most promising proteins that regulate androgen production in H295R cells. To exclude background and batch-to-batch variation, we analyzed three different batches of secretomes. As a result, we determined 26 common elements that were secreted by MSCs within 24 h and 20 proteins that were secreted within 48 h. Based on our protein profile data, we suggested a potential pathway that suppresses androgen production in H295R cells via these proteins.

When we analyzed the total secretome from MSC, we found more than 800 identified proteins. To exclude nonspecific proteins, we compared the protein profile of secretome collected from MSC with that of other somatic cells (fibroblasts). By comparing MSC secretome and fibroblast secretome, we excluded most nonspecific proteins and reported 178 MSC-specific proteins for further analysis.

After we obtained a list of the major proteins in the MSC secretome that suppress androgen synthesis, we analyzed the functional protein interaction network for the androgen suppression pathway using the data mining algorithm STRING [39,40,41]. To build a protein interaction model related to the *CYP17A1* regulation pathway, we first searched for the known *CYP17A1* inhibitor pathways in the published literature. In several published papers, it was reported that the MAPK (MEK) pathway inhibits *CYP17A1* [42,43,44]. In the STRING analysis data, we also found a direct interaction between candidate proteins in the MSC secretome (ACTB, A1BG, AHSG, APOA1, COL1A1, COL1A2, FN1, HP, HPX, ITIH4, PGLYRP2, TF and ALB) and MAPK pathway proteins such as MAP2K1, SRC, EGF and FGF2. Therefore, we suggest that the *CYP17A1* suppression pathway is induced by MSC-derived proteins through the MAPK pathway. We tried the same approach for *CYP11A1* and *DENND1A* but could not find a specific pathway known to suppress them. In the STRING analysis, more than 20 protein interactions were found between the MSC-derived proteins and our target proteins (*CYP11A1* and *DENND1A*). Further studies should be conducted to identify major regulation pathways that suppress *CYP11A1* and *DENND1A*.

Although we identified many interesting proteins in our analysis of the MSC-secretome using a data-dependent mass spectrometry approach, several other key proteins such as cytokine and exosomal proteins could have been under the limit of detection when using this approach. In various published papers, it has been reported that MSCs secrete cytokines such as bone morphogenetic proteins (BMPs) and [45,46] interleukin (IL) [47,48,49]. Our previous study also reported IL-10 secretion by hBM-MSCs [38]. In this study, we did not identify any cytokines in the EL-1 fraction of the MSC secretome; however, we did identify an insulin-like growth factor (IGF2), a cytokine, in the unfractionated MSC secretome. Therefore, we posit that some of the cytokines may have been lost during the fractionation procedure. To accommodate the missing information in this study, analyzing cytokine profiles with different methods would be an interesting topic for future study.

The major outcome of androgen synthesis regulation is the testosterone level secreted from the control and each set of elute-treated H295R cells. Unfortunately, we could not analyze testosterone levels due to the limited sample size of each elute. Although we did not analyze it directly, we can infer testosterone production through gene expression levels. In our previous study, we reported that H295R cells with decreased gene expression of *CYP17A1*, *CYP11A1* and *DENND1A* show decreased testosterone production [38]. In our future studies exploring detailed regulation pathways, we may evaluate testosterone analysis to suggest the most reliable regulatory model.

In this study, we analyzed three fractions of conditioned media to screen for major factors that regulate the expression of genes involved in androgen synthesis. We report that one of the fractions shows the highest regulatory effect on androgen synthesis genes. This fraction was analyzed through a mass spectrometry analysis to identify a common set of protein components. The protein interaction network analysis only suggested a potential pathway responsible for BMSC secretome’s effect on androgen production. However, such a hypothesis was not further investigated. Therefore, the correlation between the secreted proteins identified in this study and androgen production needs to be analyzed in further studies.

Although we analyzed three independent samples of EL-1 to minimize batch-to-batch variations, we could not test samples from multiple MSCs. Because we used MSCs from a single donor, we may have missed donor-related variations. This limitation can be overcome by using MSC from several different donors. By comparing secretomes from different donor MSCs, it is possible to exclude donor-specific proteins and make a better organized list of key proteins for androgen regulation. In addition, using MSCs from different origins (tissues) may help us to identify key regulator proteins. In future studies, analyzing these donor-related variations and origin-related variations would be an interesting topic to aid our understanding of the main therapeutic pathways of the secreted proteins.

When we analyzed the gene expression level of *CYP17A1* and *DENND1A*, we found significant change both 24 and 48 h after treatment. In particular, EL-1 treatment shows equal or better regulation effects compared to those of total CM treatment. This result indicates that proteins that coexist in both the 24 h EL-1 sample and the 48 h EL-1 sample are key factors of *CYP17A1* and *DENND1A* gene suppression. Therefore, we used the common proteins of 24 h EL-1 and 48 h EL-1 as candidates for protein interactions leading to *CYP17A1* and *DENND1A* gene suppression. On the other hand, CYP11A1 gene expression was significantly changed by elute samples only in 48-h EL treated groups. Therefore, we analyzed the unique proteins of 48-h EL-1 as candidates of protein interactions leading to *CYP11A1* gene suppression.

In this study, we suggested several candidate proteins that may regulate the androgen production pathway and expected protein interactions using STRING analysis. In future studies, we may evaluate the specific pathway of each protein and confirm intermediate protein activity in the STRING analysis, which could not be tested here due to the limited sample size. For example, we can analyze any changes in androgen synthesis genes (*CYP17A1*, *CYP11A1 DENND1A*) and activity changes in intermediate proteins such as MAPK and RAB35. This additional assay will verify the suggested pathway in this study and the main regulatory protein of androgen synthesis.

Among the candidate proteins we report, some candidate proteins look more promising than other candidate proteins reported in the literature, For example, A1BG may regulate *CYP17A1* and *DENND1A*, and it is reported that A1BG is involved in inflammatory events [50,51]. In particular, it is reported that A1BG was significantly decreased in the inflamed livers of obese mice [51]. Obesity low-grade inflammation is one of the commonly reported symptoms in PCOS and is related to high androgen production [38]. Therefore, future studies on A1BG would be interesting to understand the suppression pathway of androgen synthesis. Another candidate protein, APOA1, is also reportedly downregulated in PCOS patients’ ovaries [52]. In addition, APOA1 is related to cholesterol metabolism and steroid hormone synthesis [52,53]. Our study also suggested that APOA1 is one of the regulation factors of the androgen production genes *CYP17A1* and *DENND1A*. Therefore, APOA1 is a strong candidate for future study to understand the restoring mechanism of PCOS.

Here, we present several candidate proteins as androgen synthesis regulators. Our data suggest that MSCs secrete proteins that suppress androgen synthesis by inhibiting *CYP11A1*, *CYP17A1* and *DENND1A* gene expression. However, we did not test the effect of every single protein on the androgen synthesis pathway. Analyzing the dose-dependent effect of each protein is very important to identifying key molecules of androgen regulation and understanding the major regulatory mechanisms. In future studies, we plan to analyze each protein in more detail in order to identify androgen regulation mechanisms via MSCs. The protein profile of the hBM-MSC secretome that suppresses androgen production is a valuable resource for developing novel treatments for PCOS. For example, PCOS treatment using MSCs could be made more effective through the stimulation of certain protein secretions in MSCs. In this study, we reported several proteins in the hBM-MSC secretome that suppress androgen production. Using these candidate proteins, we may be able to develop novel treatment options for women with PCOS.

## 4. Materials and Methods

### 4.1. Human Mesenchymal Stem Cell Culture

Human bone marrow mesenchymal stem cells (MSCs, P2) purchased from RoosterBio (Cat# MSC-031, Frederick, MD, USA) were isolated from a female donor. The cells were expanded as mentioned previously [37]. In brief, the cells were treated as per the recommended cell culture medium, using RoosterNourish™-MSC-XF (RoosterBio, Frederick, MD, USA), which consisted of RoosterBasal™-MSC basal medium and RoosterBooster™-MSC supplementary reagent, as per the expansion protocol. When the culture reached about 80% confluence, the cells were passaged for two to four more serial expansions for the collection of the conditioned medium.

### 4.2. Collection of the Secretome from MSCs

The secretomes (MSC-conditioned medium, MSC-CM) from MSCs were prepared using three to five passages of mesenchymal stem cells, as mentioned previously by our group [37]. A total of 80 to 90% cell confluence flasks were used to collect the conditioned medium from the MSCs. To remove culture medium-derived proteins, the media were exchanged with RoosterBasal™-MSC basal medium without supplement reagent when cells reached around 80% confluency. The MSC-CM was collected separately in a 24-h group and a 48-h group, centrifuged at 500× *g* for 10 min at 4 °C to remove the cell debris, aliquoted and stored at −80 °C for further use. RoosterBasal™-MSC basal medium without cells was also incubated for 24 h in culture flasks for use as a negative control.

### 4.3. Human Fibroblast Culture and Secretome Collection 

Human dermal fibroblast (Fibroblast) was purchased from ATCC (Manassas, VA, USA). In brief, the cells were treated as per the recommended cell culture medium, using DMEM High glucose (Gibco, Waltham, MA, USA), with 10% FBS (Gibco, Waltham, MA, USA) and Penicillin-Streptomycin (Gibco, Waltham, MA, USA), as per the expansion protocol. When the cultured cells reached about 80% confluence, the cell media were exchanged with RoosterBasal™-MSC basal medium without the supplement reagent to prepare CM in the same condition. The cells were incubated for 24 h in culture flasks to produce the Fibroblast CM.

### 4.4. Fractionation of hBM-MSC-CM

Sephadex-15 beads (size < 1500 da) were used to fractionate molecules in the hBM-MSCs by size. Briefly, the Sephadex-15 beads were soaked in phosphate buffered saline (PBS) for 24 h to allow them to swell. Next, the bead slurry was added to a 10 mL column, where the bed volume was ~40–50% of the total column volume, providing optimal resolution of the proteins from each CM fraction. Each column was subsequently incubated at 4 °C for 24 h. Before use, the columns were packed via centrifugation at 4500× *g*. The columns were then washed with deionized water three times, followed by three PBS washes and was finally equilibrated with three washes using basal medium. MSC-derived CM was loaded into the column and the first fraction was collected without any centrifugation (gravity-based elution), where a total of 40 mL of media was passed through the column for the collection of the gravity-based elute (Elute-1). Next, the column was loaded with 20 mL of basal media and a second fraction (Elute-2) was collected after centrifugation at 4500× *g* for 5 min. Finally, another 20 mL of basal media was added and a third fraction was collected (Elute-3) after centrifugation at 4500× *g* (Figure 1). All three fractions were concentrated using 3 kDa molecular weight cutoff filters before the evaluation of androgen production activity in the H295R cells.

### 4.5. Activity-Based Analysis of hBM-MSC-CM Fractions

The human adrenocortical carcinoma cell line (H295R) was used to check the androgen production activity of all three elutes of the 24- and 48-h groups. The H295R cells were purchased from ATCC (Manassas, VA, USA) and cultured as per the recommended guidelines. Briefly, the H295R cells were cultured in flasks precoated with extracellular matrix (Gibco, Waltham, MA, USA) with DMEM/F12 (Gibco, Waltham, MA, USA) and 2.5% of Nu-Serum (Corning, Corning, NY, USA). The cells were sub-cultured at a ratio of 1:3 to 1:4, and the culture medium was changed twice a week. The H295R cells were cultured on six-well plates pre-coated with extracellular matrix at a density of 1.8 × 10^5^ cells per well for 48 h prior to treatment with the three elutes of MSC-CM collected at 24 and 48 h. The cells were treated with different elutes for 48 h. After treatment, the medium was removed and the cells were washed with PBS three times, trypsinized, and collected for analysis of steroidogenesis pathway gene expressions such as *CYP11A1*, *CYP17A1* and *DENND1A* by qPCR.

### 4.6. Quantitative RT-qPCR

RNA isolation was performed using TRIzol Reagent (Invitrogen), according to the manufacturer’s instructions as described previously [37]. The concentration and purity of RNA was quantified by spectrophotometry at 260 nm using a Nanodrop 2000 (Thermo Fisher Scientific, Waltham, MA, USA). A quantity of 1 µg of total RNA was reverse-transcribed using the RNA to cDNA EcoDry premix (Takara bio, Kusatsu, Shiga, Japan). Real-time PCR was performed using the CFX96 PCR instrument and Universal SYBR Green Supermix (Bio-Rad, Hercules, CA, USA). The list of primers including *CYP11A1*, *CYP17A1* and *DENND1A* are available in Supplementary Table S2. The following PCR parameters were used: initial denaturation cycle at 95 °C for 3 min, followed by 40 amplification cycles at 95 °C for 10 s, 56 °C for 15 s, and 72 °C for 1 min. The results are presented as the fold change in relative gene expression quantified using the delta–delta CT(ΔΔCt) method. Beta-actin was used as a reference gene for sample normalization.

### 4.7. Mass Spectrometry Analysis of MSC-CM

Protein concentration for each CM sample was determined via a bicinchoninic acid assay. Next, 100 μg of protein from each sample was subjected to reduction with 10 mM dithiothreitol for 20 min at 55 °C and alkylated with 30 mM iodoacetamide for 20 min at room temperature, followed by trysin digestion (1:20 wt/wt trypsin:protein). Peptides from each sample were isotopically labelled using a TMT-6 plex kit and fractionated via reversed-phase high pH chromatography. Peptide separation and mass detection occurred using a Q-Exactive mass spectrometer as previously described [54]. Raw data for the liquid chromatography–mass spectrometry (LC–MS) analysis were searched against the Swiss Protein *Homosapien* database using the Proteome Discoverer (v2.3, Thermo Fisher, Carlsbad, CA, USA) software. Here, trypsin was set as the protease with two missed cleavages and searches were performed with precursor and fragment mass error tolerances set to 10 ppm and 0.02 Da, respectively. Peptide variable modifications allowed during the search were oxidation (M) and TMT 6-plex (S, T, Y), whereas carbamidomethyl (C) and TMT 6-plex (peptide N-terminus and (K)) were set as fixed modifications. Differentially expressed proteins for MSC relative to fibroblasts was determined by applying an unpaired *t*-test (*p* ≤ 0.05). In a similar manner, proteins in the EL-1 MSC-CM sample were identified; however, fractionation of the proteome was not performed prior to LC–MS analysis. The peptide variable modification allowed during the search was oxidation (M), whereas carbamidomethyl (C) was used as a fixed modification.

### 4.8. Protein–Protein Interaction Analysis

The Search Tool for protein-to-protein interaction String Analysis (STRINGv11.5) was used [55,56]. The network was set for common proteins in three independent replicates of EL-1 samples using a median confidence level (CL) of 0.40 and shows direct reactions up to 20 interactors. 

### 4.9. Statistical Analysis

Comparisons between groups were made by two-way ANOVA using GraphPad Prism 9 (GraphPad Software, San Diego, CA, USA). A difference between groups of *p* < 0.05 was considered significant.

## Figures and Tables

**Table 1:** Twenty-six common elements in “Elute24h_A”, “Elute24h_B” and “Elute24h_C”.

	Description
1	Actin, cytoplasmic 1 OS = Homo sapiens OX = 9606 GN = ACTB PE = 1 SV = 1
2	Alpha-1B-glycoprotein OS = Homo sapiens OX = 9606 GN = A1BG PE = 1 SV = 4
3	Alpha-2-HS-glycoprotein OS = Homo sapiens OX = 9606 GN = AHSG PE = 1 SV = 2
4	Apolipoprotein A-I OS = Homo sapiens OX = 9606 GN = APOA1 PE = 1 SV = 1
5	Beta-2-glycoprotein 1 OS = Homo sapiens OX = 9606 GN = APOH PE = 1 SV = 3
6	Collagen alpha-1(I) chain OS = Homo sapiens OX = 9606 GN = COL1A1 PE = 1 SV = 5
7	Collagen alpha-2(I) chain OS = Homo sapiens OX = 9606 GN = COL1A2 PE = 1 SV = 7
8	Fibrillin-2 OS = Homo sapiens OX = 9606 GN = FBN2 PE = 1 SV = 3
9	Fibronectin OS = Homo sapiens OX = 9606 GN = FN1 PE = 1 SV = 4
10	Fibulin-1 OS = Homo sapiens OX = 9606 GN = FBLN1 PE = 1 SV = 4
11	Fibulin-5 OS = Homo sapiens OX = 9606 GN = FBLN5 PE = 1 SV = 1
12	Haptoglobin OS = Homo sapiens OX = 9606 GN = HP PE = 1 SV = 1
13	Haptoglobin-related protein OS = Homo sapiens OX = 9606 GN = HPR PE = 2 SV = 2
14	Hemoglobin subunit alpha OS = Homo sapiens OX = 9606 GN = HBA1 PE = 1 SV = 2
15	Hemopexin OS = Homo sapiens OX = 9606 GN = HPX PE = 1 SV = 2
16	Immunoglobulin heavy constant gamma 3 OS = Homo sapiens OX = 9606 GN = IGHG3 PE = 1 SV = 2
17	Insulin-like growth factor-binding protein 4 OS = Homo sapiens OX = 9606 GN = IGFBP4 PE = 1 SV = 2
18	Insulin-like growth factor-binding protein 6 OS = Homo sapiens OX = 9606 GN = IGFBP6 PE = 1 SV = 1
19	Inter-alpha-trypsin inhibitor heavy chain H4 OS = Homo sapiens OX = 9606 GN = ITIH4 PE = 1 SV = 4
20	Isoform LMW of Kininogen-1 OS = Homo sapiens OX = 9606 GN = KNG1
21	Latent-transforming growth factor beta-binding protein 2 OS = Homo sapiens OX = 9606 GN = LTBP2 PE = 1 SV = 3
22	Metalloproteinase inhibitor 1 OS = Homo sapiens OX = 9606 GN = TIMP1 PE = 1 SV = 1
23	N-acetylmuramoyl-L-alanine amidase OS = Homo sapiens OX = 9606 GN = PGLYRP2 PE = 1 SV = 1
24	Serotransferrin OS = Homo sapiens OX = 9606 GN = TF PE = 1 SV = 3
25	Serum albumin OS = Homo sapiens OX = 9606 GN = ALB PE = 1 SV = 2
26	Thrombospondin-2 OS = Homo sapiens OX = 9606 GN = THBS2 PE = 1 SV = 2

**Table 2:** Twenty common elements in “Elute48h_A”, “Elute48h_B” and “Elute48h_C”.

	Description
1	Actin, cytoplasmic 1 OS = Homo sapiens OX = 9606 GN = ACTB PE = 1 SV = 1
2	Afamin OS = Homo sapiens OX = 9606 GN = AFM PE = 1 SV = 1
3	Alpha-1B-glycoprotein OS = Homo sapiens OX = 9606 GN = A1BG PE = 1 SV = 4
4	Alpha-2-HS-glycoprotein OS = Homo sapiens OX = 9606 GN = AHSG PE = 1 SV = 2
5	Apolipoprotein A-I OS = Homo sapiens OX = 9606 GN = APOA1 PE = 1 SV = 1
6	Clusterin OS = Homo sapiens OX = 9606 GN = CLU PE = 1 SV = 1
7	Collagen alpha-1(I) chain OS = Homo sapiens OX = 9606 GN = COL1A1 PE = 1 SV = 5
8	Collagen alpha-1(III) chain OS = Homo sapiens OX = 9606 GN = COL3A1 PE = 1 SV = 4
9	Collagen alpha-1(VI) chain OS = Homo sapiens OX = 9606 GN = COL6A1 PE = 1 SV = 3
10	Collagen alpha-2(I) chain OS = Homo sapiens OX = 9606 GN = COL1A2 PE = 1 SV = 7
11	Collagen alpha-2(V) chain OS = Homo sapiens OX = 9606 GN = COL5A2 PE = 1 SV = 3
12	Decorin OS = Homo sapiens OX = 9606 GN = DCN PE = 1 SV = 1
13	Fibronectin OS = Homo sapiens OX = 9606 GN = FN1 PE = 1 SV = 4
14	Growth arrest-specific protein 6 OS = Homo sapiens OX = 9606 GN = GAS6 PE = 1 SV = 3
15	Haptoglobin OS = Homo sapiens OX = 9606 GN = HP PE = 1 SV = 1
16	Hemopexin OS = Homo sapiens OX = 9606 GN = HPX PE = 1 SV = 2
17	Inter-alpha-trypsin inhibitor heavy chain H4 OS = Homo sapiens OX = 9606 GN = ITIH4 PE = 1 SV = 4
18	N-acetylmuramoyl-L-alanine amidase OS = Homo sapiens OX = 9606 GN = PGLYRP2 PE = 1 SV = 1
19	Serotransferrin OS = Homo sapiens OX = 9606 GN = TF PE = 1 SV = 3
20	Serum albumin OS = Homo sapiens OX = 9606 GN = ALB PE = 1 SV = 2

## Supplementary Materials

The following supporting information can be downloaded at: https://www.mdpi.com/article/10.3390/ijms23094633/s1.
