# Supplementary material for: Non-Cytokine Protein Profile of the Mesenchymal Stem Cell Secretome That Regulates the Androgen Production Pathway
Source: Int J Mol Sci. 2022 Apr 22;23(9):4633. doi: 10.3390/ijms23094633 (PMC9101816; doi:10.3390/ijms23094633)
Supplement: Supplementary file 1 [file ijms-23-04633-s001.zip › ijms-1670592-supplementary.pdf]

Supplementary Table S1. Altered Protein profile total MSC conditioned media.

Description	Sum PEP Score	Coverage [%]	MW [kDa]	Abundance Ratio: (Sample / Control)	Abundance Ratio Adj. P-Value
**Angiopoietin-2 OS=Homo sapiens GN=ANGPT2 PE=1 SV=1**	6.262	6	56.9	11.333	0.086016647
**Hyaluronan and proteoglycan link protein 1 OS=Homo sapiens GN=HAPLN1 PE=2 SV=2**	48.902	29	40.1	8.549	0.064898916
**Carboxypeptidase A4 OS=Homo sapiens GN=CPA4 PE=1 SV=2**	30.125	17	47.3	7.553	0.047364885
**Serglycin OS=Homo sapiens GN=SRGN PE=1 SV=3**	55.725	39	17.6	6.639	0.037023471
**Alpha-2-HS-glycoprotein OS=Homo sapiens GN=AHSG PE=1 SV=1**	19.152	7	39.4	6.204	0.083869466
**Insulin-like growth factor-binding protein 7 OS=Homo sapiens GN=IGFBP7 PE=1 SV=1**	211.289	61	29.1	5.569	0.059417864
**Proenkephalin-A OS=Homo sapiens GN=PENK PE=1 SV=1**	32.579	23	30.8	5.472	0.054622459
**Protein HP-20 homolog OS=Bos taurus PE=2 SV=1**	18.692	19	20.6	5.269	0.053527124
**Insulin-like growth factor-binding protein 5 OS=Homo sapiens GN=IGFBP5 PE=1 SV=1**	66.056	52	30.6	5.122	0.040098658
**Apolipoprotein A-II OS=Bos taurus GN=APOA2 PE=1 SV=2**	39.568	43	11.2	5.033	0.059417864
**Insulin-like growth factor-binding protein 2 OS=Homo sapiens GN=IGFBP2 PE=1 SV=2**	97.513	48	34.8	4.785	0.052795147
**EGF-like repeat and discoidin I-like domain-containing protein 3 OS=Homo sapiens GN=EDIL3 PE=1 SV=1**	34.401	20	53.7	4.75	0.047356137
**Thrombospondin-1 (Fragment) OS=Bos taurus PE=2 SV=1**	146.73	70	25	4.36	0.077312745
**Signal peptide, CUB and EGF-like domain-containing protein 3 OS=Homo sapiens GN=SCUBE3 PE=1 SV=1**	26.842	12	109.2	4.306	0.054666294
**40S ribosomal protein S15a OS=Homo sapiens GN=RPS15A PE=1 SV=2**	4.433	26	14.8	4.058	0.104469424

**Angiotensinogen OS=Bos taurus GN=AGT PE=2 SV=1**	71.919	26	45.4	4.047	0.083418924
**Retinol-binding protein 4 OS=Homo sapiens GN=RBP4 PE=1 SV=3**	11.259	9	23	4.04	0.076036742
**Alpha-2-HS-glycoprotein OS=Bos taurus GN=AHSG PE=1 SV=2**	536.213	70	38.4	3.863	0.099319929
**(Bos taurus) 54 kDa protein [OS=Bos taurus]**	407.115	58	53.6	3.71	0.083387977
**Complement factor B (Fragment) OS=Bos taurus GN=BF PE=4 SV=1**	23.458	38	9.1	3.681	0.06751879
**Integral membrane protein 2B OS=Homo sapiens GN=ITM2B PE=1 SV=1**	4.678	7	30.3	3.674	0.047364885
**SERPIND1 protein OS=Bos taurus GN=SERPIND1 PE=2 SV=1**	83.144	31	55.2	3.66	0.049935383
**SERPINA10 protein OS=Bos taurus GN=SERPINA10 PE=2 SV=1**	16.47	8	52	3.64	0.066748056
**Exportin-1 OS=Homo sapiens GN=XPO1 PE=1 SV=1**	9.737	3	123.3	3.637	0.064898916
**Alpha-1-acid glycoprotein OS=Bos taurus GN=ORM1 PE=2 SV=1**	240.539	59	23.2	3.59	0.071992388
**Thrombospondin-4 OS=Homo sapiens GN=THBS4 PE=1 SV=2**	37.86	10	105.8	3.562	0.067696091
**Apolipoprotein A-I OS=Bos taurus GN=APOA1 PE=1 SV=3**	325.409	82	30.3	3.528	0.104469424
**Serpin A3-1 OS=Bos taurus GN=SERPINA3-1 PE=1 SV=3**	103.595	40	46.2	3.512	0.093577566
**Protein AMBP OS=Bos taurus GN=AMBP PE=1 SV=2**	132.777	36	39.2	3.505	0.083387977
**Complement component C5a (Fragment) OS=Bos taurus PE=2 SV=1**	12.93	50	8.5	3.439	0.104469424
**Factor XIIa inhibitor OS=Bos taurus PE=1 SV=1**	28.542	12	51.7	3.409	0.103940476
**Inter-alpha-trypsin inhibitor heavy chain H4 OS=Bos taurus GN=ITIH4 PE=1 SV=1**	201.764	33	101.4	3.345	0.080994635
**Alpha-2-antiplasmin OS=Bos taurus GN=SERPINF2 PE=1 SV=2**	117.779	29	54.7	3.342	0.064898916
**Antithrombin-III OS=Bos taurus GN=SERPINC1 PE=1 SV=2**	199.336	53	52.3	3.308	0.071767666
**Fetuin-B OS=Bos taurus GN=FETUB PE=2 SV=1**	308.162	47	42.6	3.304	0.059417864

**Connective tissue growth factor OS=Homo sapiens GN=CTGF PE=1 SV=2**	79.949	51	38.1	3.303	0.058698427
**Serum albumin OS=Bos taurus GN=ALB PE=1 SV=4**	1817.058	87	69.2	3.303	0.067501413
**(Bos taurus) similar to Complement C4-A precursor [OS=Bos taurus]**	257.262	32	192.9	3.287	0.101595024
**Chitinase-3-like protein 1 OS=Homo sapiens GN=CHI3L1 PE=1 SV=2**	14.179	13	42.6	3.275	0.047356137
**Inter-alpha-trypsin inhibitor heavy chain H3 OS=Homo sapiens GN=ITIH3 PE=1 SV=2**	24.939	5	99.8	3.274	0.066748056
**Prothrombin OS=Bos taurus GN=F2 PE=1 SV=2**	219.885	50	70.5	3.253	0.067501413
**Insulin-like growth factor-binding protein 4 OS=Homo sapiens GN=IGFBP4 PE=1 SV=2**	135.293	46	27.9	3.225	0.064898916
**Vitamin D-binding protein OS=Bos taurus GN=GC PE=2 SV=1**	428.081	62	53.3	3.209	0.080994635
**(Bos taurus) similar to fibulin-1 C isoform 1 [OS=Bos taurus]**	174.169	37	77.4	3.204	0.059417864
**Serotransferrin OS=Bos taurus GN=TF PE=2 SV=1**	1768.648	87	77.7	3.176	0.053527124
**Ras-related protein Rab-5A OS=Homo sapiens GN=RAB5A PE=1 SV=2**	11.64	15	23.6	3.167	0.115351174
**Complement component C9 OS=Bos taurus GN=C9 PE=2 SV=1**	42.33	26	62	3.148	0.067696091
**Tyrosine-protein kinase receptor UFO OS=Homo sapiens GN=AXL PE=1 SV=3**	9.291	3	98.3	3.146	0.071992388
**Thyroxine-binding globulin OS=Bos taurus GN=SERPINA7 PE=2 SV=1**	60.602	27	46	3.124	0.080068185
**Polypeptide N-acetylgalactosaminyltransferase 5 OS=Homo sapiens GN=GALNT5 PE=1 SV=1**	10.155	5	106.2	3.115	0.047356137
**Pigment epithelium-derived factor OS=Bos taurus GN=SERPINF1 PE=1 SV=1**	89.419	33	46.2	3.096	0.096141268
**Lysyl oxidase homolog 2 OS=Homo sapiens GN=LOXL2 PE=1 SV=1**	103.801	26	86.7	3.093	0.062726234

**Cell migration-inducing and hyaluronan-binding protein OS=Homo sapiens GN=CEMIP PE=1 SV=2**	25.064	7	152.9	3.086	0.080068185
**ApoN protein OS=Bos taurus GN=ApoN PE=2 SV=1**	12.041	22	28.5	3.081	0.083869466
**Cadherin-6 OS=Homo sapiens GN=CDH6 PE=1 SV=1**	10.89	5	88.3	3.077	0.067696091
**Plasma serine protease inhibitor OS=Bos taurus GN=SERPINA5 PE=1 SV=1**	52.394	29	45.3	3.069	0.078234816
**HLA class I histocompatibility antigen, A-3 alpha chain OS=Homo sapiens GN=HLA-A PE=1 SV=1**	9.469	9	44.4	3.066	0.028004287
**Major prion protein OS=Homo sapiens GN=PRNP PE=1 SV=1**	10.007	8	27.6	3.05	0.082593959
**(Bos taurus) similar to C4b-binding protein alpha chain [OS=Bos taurus]**	40.507	60	22.3	3.043	0.064898916
**Complement C3 OS=Bos taurus GN=C3 PE=1 SV=2**	649.349	52	187.1	3.017	0.100034252
**Neogenin OS=Homo sapiens GN=NEO1 PE=1 SV=2**	5.269	2	159.9	3.005	0.437286512
**Hemoglobin subunit beta OS=Bos taurus GN=HBB PE=1 SV=1**	22.351	20	15.9	2.993	0.137514708
**Alpha-1B-glycoprotein OS=Bos taurus GN=A1BG PE=1 SV=1**	210.369	55	53.5	2.986	0.06751879
**Inter-alpha-trypsin inhibitor heavy chain H2 OS=Homo sapiens GN=ITIH2 PE=1 SV=2**	60.865	7	106.4	2.978	0.073244736
**A disintegrin and metalloproteinase with thrombospondin motifs 2 OS=Homo sapiens GN=ADAMTS2 PE=2 SV=2**	9.394	4	134.7	2.975	0.105281478
**Stanniocalcin-2 OS=Homo sapiens GN=STC2 PE=1 SV=1**	48.604	34	33.2	2.97	0.06634983
**Biglycan OS=Homo sapiens GN=BGN PE=1 SV=2**	224.183	51	41.6	2.967	0.083387977
**Plasminogen OS=Bos taurus GN=PLG PE=1 SV=2**	405.856	73	91.2	2.96	0.067696091
**Proprotein convertase subtilisin/kexin type 9 OS=Homo sapiens GN=PCSK9 PE=1 SV=3**	7.018	4	74.2	2.907	0.071992388

**Regucalcin OS=Homo sapiens GN=RGN PE=1 SV=1**	9.749	9	33.2	2.881	0.136763538
**Alpha-amylase OS=Bos taurus GN=AMY2A PE=2 SV=1**	19.861	15	57.4	2.879	0.072311413
**(Bos taurus) 55 kDa protein [OS=Bos taurus]**	20.852	25	54.6	2.862	0.150309214
**(Bos taurus) similar to Pregnancy zone protein, partial [OS=Bos taurus]**	60.824	23	65.2	2.859	0.080994635
**Beta-2-glycoprotein 1 OS=Bos taurus GN=APOH PE=1 SV=4**	155.558	48	38.2	2.859	0.067501413
**Serpin A3-4 OS=Bos taurus GN=SERPINA3-4 PE=3 SV=1**	110.03	39	46.3	2.858	0.078234816
**Clusterin OS=Bos taurus GN=CLU PE=1 SV=1**	98.7	37	51.1	2.836	0.092134553
**(Bos taurus) similar to afamin [OS=Bos taurus]**	164.575	39	69.5	2.817	0.071992388
**Acidic mammalian chitinase OS=Bos taurus GN=CHIA PE=1 SV=1**	27.644	15	52.1	2.762	0.080976111
**Uncharacterized protein (Fragment) OS=Bos taurus GN=HGFAC PE=3 SV=2**	24.485	21	32.7	2.76	0.078234816
**Inter-alpha-trypsin inhibitor heavy chain H1 OS=Bos taurus GN=ITIH1 PE=2 SV=1**	95.315	18	101.2	2.756	0.071767666
**(Bos taurus) similar to endopin 2B [OS=Bos taurus]**	134.587	29	46.9	2.752	0.081827115
**(Bos taurus) 15 kDa protein [OS=Bos taurus]**	8.191	35	14.8	2.734	0.078234816
**Complement C4 (Fragments) OS=Bos taurus GN=C4 PE=1 SV=2**	122.072	31	101.8	2.724	0.124235574
**Pregnancy zone protein OS=Homo sapiens GN=PZP PE=1 SV=4**	28.805	4	163.8	2.721	0.064898916
**Complement factor H OS=Homo sapiens GN=CFH PE=1 SV=4**	60.343	17	139	2.703	0.080976111
**Apolipoprotein A-IV OS=Bos taurus GN=APOA4 PE=2 SV=1**	49.37	30	43	2.679	0.096141268
**Leucine-rich alpha-2-glycoprotein 1 OS=Bos taurus GN=LRG1 PE=2 SV=1**	45.798	27	38.3	2.675	0.06751879
**Complement component C7 OS=Bos taurus GN=C7 PE=2 SV=1**	116.276	35	93	2.674	0.076514553
**Beta-casein OS=Bos taurus GN=CSN2 PE=1 SV=2**	16.343	15	25.1	2.668	0.44977794

**Complement factor H OS=Bos taurus GN=CFH PE=1 SV=3**	231.817	40	140.3	2.668	0.064898916
**Cadherin-13 OS=Homo sapiens GN=CDH13 PE=1 SV=1**	15.999	7	78.2	2.64	0.083418924
**Complement factor I OS=Bos taurus GN=CFI PE=2 SV=1**	119.761	43	68.9	2.634	0.062726234
**Poliovirus receptor OS=Homo sapiens GN=PVR PE=1 SV=1**	8.165	6	45.3	2.607	0.059417864
**Carboxypeptidase N catalytic chain OS=Bos taurus GN=CPN1 PE=2 SV=1**	11.645	10	52.6	2.604	0.083869466
**Cartilage oligomeric matrix protein OS=Bos taurus GN=COMP PE=4 SV=2**	105.026	29	81.8	2.6	0.073244736
**Alpha-1-antiproteinase OS=Bos taurus GN=SERPINA1 PE=1 SV=1**	547.814	58	46.1	2.599	0.066373808
**Periostin variant 7 OS=Bos taurus GN=POSTN PE=2 SV=1**	121.177	35	86.8	2.594	0.115196307
**Dystroglycan OS=Homo sapiens GN=DAG1 PE=1 SV=2**	31.245	8	97.4	2.592	0.105281478
**Plasma kallikrein (Fragment) OS=Homo sapiens GN=KLKB1 PE=1 SV=1**	6.01	1	76.8	2.585	0.064898916
**Fibrinogen alpha chain OS=Bos taurus GN=FGA PE=1 SV=5**	138.762	37	67	2.547	0.080976111
**Heterogeneous nuclear ribonucleoprotein U-like protein 2 OS=Homo sapiens GN=HNRNPUL2 PE=1 SV=1**	6.087	3	85.1	2.533	0.170933315
**Coiled-coil domain-containing protein 80 OS=Homo sapiens GN=CCDC80 PE=1 SV=1**	96.68	25	108.1	2.532	0.084077711
**Serum albumin OS=Homo sapiens GN=ALB PE=1 SV=2**	110.096	19	69.3	2.524	0.143979347
**Apolipoprotein E OS=Bos taurus GN=APOE PE=2 SV=1**	66.73	51	36	2.52	0.104469424
**Membrane primary amine oxidase OS=Homo sapiens GN=AOC3 PE=1 SV=3**	5.404	2	84.6	2.49	0.080976111
**Thrombospondin-3 OS=Homo sapiens GN=THBS3 PE=1 SV=1**	47.06	13	104.1	2.489	0.089731259
**Procollagen-lysine,2-oxoglutarate 5-dioxygenase 1 OS=Homo sapiens GN=PLOD1 PE=1 SV=2**	74.218	23	83.5	2.488	0.108390635
**Alpha-fetoprotein OS=Bos taurus GN=AFP PE=2 SV=1**	733.54	76	68.5	2.477	0.090515344

**Carboxypeptidase B2 OS=Bos taurus GN=CPB2 PE=1 SV=1**	14.17	11	48.8	2.475	0.064898916
**Sushi repeat-containing protein SRPX2 OS=Homo sapiens GN=SRPX2 PE=1 SV=1**	10.41	9	52.9	2.469	0.083830482
**Nascent polypeptide-associated complex subunit alpha, muscle-specific form OS=Homo sapiens GN=NACA PE=1 SV=1**	9.945	1	205.3	2.463	0.143979347
**Sulfhydryl oxidase 1 OS=Homo sapiens GN=QSOX1 PE=1 SV=3**	120.018	34	82.5	2.451	0.102018238
**Serotransferrin-like OS=Bos taurus GN=LOC525947 PE=2 SV=1**	105.075	38	69.1	2.443	0.080519492
**Matrin-3 OS=Homo sapiens GN=MATR3 PE=1 SV=1**	10.455	5	99.9	2.434	0.113598193
**(Bos taurus) similar to alpha-2-macroglobulin isoform 1 [OS=Bos taurus]**	1084.417	62	164.2	2.433	0.105281478
**(Bos taurus) 121 kDa protein [OS=Bos taurus]**	44.938	14	121.4	2.432	0.079190829
**Galectin-3-binding protein OS=Homo sapiens GN=LGALS3BP PE=1 SV=1**	93.294	27	65.3	2.405	0.081354007
**(Bos taurus) similar to apolipoprotein B, partial [OS=Bos taurus]**	167.02	41	92.2	2.358	0.113598193
**Cadherin-11 OS=Homo sapiens GN=CDH11 PE=2 SV=2**	19.631	10	87.9	2.356	0.066748056
**Glial fibrillary acidic protein OS=Homo sapiens GN=GFAP PE=1 SV=1**	14.731	9	49.9	2.356	0.140126054
**Latent-transforming growth factor beta-binding protein 3 OS=Homo sapiens GN=LTBP3 PE=1 SV=4**	11.293	4	139.3	2.354	0.150086044
**C-type mannose receptor 2 OS=Homo sapiens GN=MRC2 PE=1 SV=2**	9.137	3	166.6	2.351	0.080976111
**Complement C5 OS=Homo sapiens GN=C5 PE=1 SV=4**	17.457	3	188.2	2.313	0.11714316
**Aggrecan core protein OS=Homo sapiens GN=ACAN PE=1 SV=2**	92.417	9	250	2.303	0.080994635
**Semaphorin-7A OS=Homo sapiens GN=SEMA7A PE=1 SV=1**	108.024	37	74.8	2.294	0.078234816

**C4b-binding protein alpha chain OS=Bos taurus GN=C4BPA PE=2 SV=1**	62.779	33	68.8	2.289	0.078234816
**Uncharacterized protein OS=Bos taurus GN=VTN PE=2 SV=1**	47.756	22	53.5	2.286	0.083387977
**Thrombospondin-1 OS=Homo sapiens GN=THBS1 PE=1 SV=2**	628.733	57	129.3	2.267	0.083387977
**EH domain-containing protein 1 OS=Homo sapiens GN=EHD1 PE=1 SV=1**	5.464	4	61.9	2.262	0.18113831
**Coagulation factor V OS=Bos taurus GN=F5 PE=1 SV=1**	44.927	6	248.8	2.262	0.105281478
**Spectrin beta chain, erythrocytic OS=Homo sapiens GN=SPTB PE=1 SV=5**	12.383	2	246.3	2.258	0.147269594
**Tubulin-specific chaperone A OS=Homo sapiens GN=TBCA PE=1 SV=1**	7.264	17	14.3	2.254	0.186907929
**Collagen and calcium-binding EGF domain-containing protein 1 OS=Homo sapiens GN=CCBE1 PE=1 SV=1**	10.677	6	44.1	2.252	0.094653191
**Thrombospondin-2 OS=Homo sapiens GN=THBS2 PE=1 SV=2**	247.593	31	129.9	2.243	0.081827115
**Olfactomedin-like protein 3 OS=Homo sapiens GN=OLFML3 PE=2 SV=1**	72.286	33	46	2.237	0.128351268
**Beta-lactoglobulin OS=Bos taurus GN=LGB PE=1 SV=3**	19.542	26	19.9	2.227	0.340678358
**Syndecan-4 OS=Homo sapiens GN=SDC4 PE=1 SV=2**	9.352	18	21.6	2.221	0.088384996
**Vitamin K-dependent protein S OS=Bos taurus GN=PROS1 PE=1 SV=1**	37.99	12	75.1	2.22	0.08938092
**Hemopexin OS=Bos taurus GN=HPX PE=2 SV=1**	168.945	53	52.2	2.212	0.105281478
**Cysteine-rich motor neuron 1 protein OS=Homo sapiens GN=CRIM1 PE=1 SV=1**	35.449	13	113.7	2.206	0.102018238
**Dynactin subunit 2 OS=Homo sapiens GN=DCTN2 PE=1 SV=4**	5.388	9	44.2	2.205	0.344046227
**Follistatin OS=Homo sapiens GN=FST PE=1 SV=2**	52.858	51	38	2.203	0.105281478
**Tropomyosin alpha-4 chain (Fragment) OS=Homo sapiens GN=TPM4 PE=1 SV=1**	6.861	29	8	2.192	0.094967147

**Prohibitin OS=Homo sapiens GN=PHB PE=1 SV=1**	12.491	13	29.8	2.184	0.09767256
**Serine/threonine-protein phosphatase PP1-alpha catalytic subunit OS=Homo sapiens GN=PPP1CA PE=1 SV=1**	17.339	15	37.5	2.184	0.096141268
**ATP-dependent 6-phosphofructokinase, platelet type OS=Homo sapiens GN=PFKP PE=1 SV=2**	7.379	5	85.5	2.176	0.251240961
**Bone morphogenetic protein 1 OS=Homo sapiens GN=BMP1 PE=1 SV=2**	38.978	13	111.2	2.172	0.080976111
**Fibrinogen beta chain OS=Bos taurus GN=FGB PE=1 SV=2**	32.403	26	53.3	2.171	0.108390635
**Transcription intermediary factor 1-beta OS=Homo sapiens GN=TRIM28 PE=1 SV=5**	5.643	4	88.5	2.169	0.120272257
**Metallothionein-1E OS=Homo sapiens GN=MT1E PE=1 SV=1**	18.605	34	6	2.162	0.080976111
**Glycerol-3-phosphate dehydrogenase [NAD(+)], cytoplasmic OS=Homo sapiens GN=GPD1 PE=1 SV=4**	13.763	6	37.5	2.161	0.084572846
**Apolipoprotein M OS=Homo sapiens GN=APOM PE=1 SV=2**	4.953	8	21.2	2.159	0.11747929
**Hemoglobin subunit alpha OS=Bos taurus GN=HBA PE=1 SV=2**	57.779	44	15.2	2.15	0.104469424
**Cadherin-2 OS=Homo sapiens GN=CDH2 PE=1 SV=4**	47.246	15	99.7	2.147	0.143803666
**Thymosin beta-10 OS=Homo sapiens GN=TMSB10 PE=1 SV=2**	20.223	32	5	2.137	0.104625132
**Insulin-like growth factor II OS=Homo sapiens GN=IGF2 PE=1 SV=1**	8.382	14	20.1	2.107	0.071992388
**Casein kinase II subunit alpha OS=Homo sapiens GN=CSNK2A1 PE=1 SV=1**	10.163	13	45.1	2.106	0.165154658
**Tetranectin OS=Homo sapiens GN=CLEC3B PE=1 SV=3**	53.094	43	22.5	2.101	0.083387977
**Guanine nucleotide-binding protein G(I)/G(S)/G(T) subunit beta-2 OS=Homo sapiens GN=GNB2 PE=1 SV=3**	31.468	29	37.3	2.1	0.236685417
**Peroxidasin homolog OS=Homo sapiens GN=PXDN PE=1 SV=2**	92.379	19	165.2	2.098	0.105281478

**Alpha-S2-casein OS=Bos taurus GN=CSN1S2 PE=1 SV=2**	16.139	14	26	2.081	0.471901593
**Fibronectin type III domain-containing protein 1 OS=Homo sapiens GN=FNDC1 PE=2 SV=4**	5.341	2	205.4	2.069	0.096141268
**Adiponectin OS=Bos taurus GN=ADIPOQ PE=1 SV=1**	52.145	29	26.1	2.069	0.104469424
**Extracellular matrix protein 2 OS=Homo sapiens GN=ECM2 PE=2 SV=1**	8.424	3	79.7	2.067	0.150086044
**Inhibin beta A chain OS=Homo sapiens GN=INHBA PE=1 SV=2**	28.251	24	47.4	2.064	0.108094343
**Dynamin-1-like protein OS=Homo sapiens GN=DNM1L PE=1 SV=2**	17.855	4	81.8	2.06	0.534393143
**Disintegrin and metalloproteinase domain-containing protein 9 OS=Homo sapiens GN=ADAM9 PE=1 SV=1**	34.445	8	90.5	2.059	0.096141268
**Pentraxin-related protein PTX3 OS=Homo sapiens GN=PTX3 PE=1 SV=3**	175.434	42	41.9	2.058	0.104469424
**Cation-independent mannose-6-phosphate receptor OS=Homo sapiens GN=IGF2R PE=1 SV=3**	12.497	2	274.2	2.055	0.115617561
**Vasorin OS=Homo sapiens GN=VASN PE=1 SV=1**	64.178	16	71.7	2.046	0.103996332
**Phosphoserine aminotransferase OS=Homo sapiens GN=PSAT1 PE=1 SV=2**	11.243	8	40.4	2.042	0.137227272
**Tubulin alpha chain (Fragment) OS=Homo sapiens GN=TUBA8 PE=1 SV=1**	111.661	34	52	2.035	0.11747929
**Tubulin beta-2B chain OS=Homo sapiens GN=TUBB2B PE=1 SV=1**	141.559	52	49.9	2.035	0.081827115
**Apolipoprotein C-III OS=Homo sapiens GN=APOC3 PE=1 SV=1**	15.83	16	12.8	2.031	0.104469424
**Glutathione reductase, mitochondrial OS=Homo sapiens GN=GSR PE=1 SV=2**	6.318	5	56.2	2.025	0.094230369
**Heterogeneous nuclear ribonucleoprotein Q OS=Homo sapiens GN=SYNCRIP PE=1 SV=2**	7.417	5	69.6	2.017	0.232275714
**Uncharacterized protein OS=Homo sapiens PE=3 SV=1**	33.403	21	23.3	2.007	0.18026907
**SPARC-like protein 1 OS=Homo sapiens GN=SPARCL1 PE=1 SV=2**	5.612	3	75.2	2.001	0.194371613

Supplementary Table S2. List of Primers for PCR.

Gene	Forward primer (5′-3′)	Reverse primer (5′-3′)
Human *ACTB*	TGGATCAGCAAGCAGGAGTATG	GCATTTGCGGTGGACGAT
Human *CYP17A1*	GGCCTCAAATGGCAACTCTAGA	CTTCTGATCGCCATCCTTGAA
Human *CYP11A1*	GAGGGAGACGGGCACACA	TGACATAAACCGACTCCACGTT
Human *DENND1A*	CAATTCCCGGAGGACTACAGT	AGCACGAATGTGAAGTTCTGG
